# Effect of etanercept in polymyalgia rheumatica: a randomized controlled trial

**DOI:** 10.1186/ar3140

**Published:** 2010-09-20

**Authors:** Frederik Kreiner, Henrik Galbo

**Affiliations:** 1 Department of Rheumatology, Institute of Inflammation Research, Rigshospitalet, Copenhagen University Hospital, Blegdamsvej 9, DK-2100 Copenhagen, Denmark

## Abstract

**Introduction:**

To elucidate in polymyalgia rheumatica (PMR) the role of tumor necrosis factor (TNF) α and the therapeutic potential of blockade with soluble TNF-α receptor, we carried out the first randomized controlled trial with etanercept in PMR.

**Methods:**

Twenty newly diagnosed, glucocorticoid (GC) naïve patients with PMR and 20 matched non-PMR control subjects completed the trial. Subjects were randomized in a 1:1 ratio to monotherapy with etanercept (25 mg s.c. biweekly) or placebo (saline) for 14 days. Study outcomes were assessed at baseline and after 14 days. The primary outcome was the change in PMR activity score (PMR-AS). Secondary outcomes were: changes in erythrocyte sedimentation rate (ESR) and plasma levels of TNF-α and interleukin (IL) 6; patients' functional status (health assessment questionnaire) and cumulative tramadol intake during the trial.

**Results:**

At baseline, plasma TNF-α was higher in patients than in controls (*P *< 0.05). The concentration always increased with etanercept treatment (*P *< 0.05). In patients, etanercept decreased PMR-AS by 24% (*P *= 0.011), reflecting significant improvements in shoulder mobility, physician's global assessment and C-reactive protein, and insignificant (*P *> 0.05) improvements in duration of morning stiffness and patient's assessment of pain. In parallel, ESR and IL-6 were reduced (*P *< 0.05). Placebo treatment did not change PMR-AS, ESR and IL-6 (*P *> 0.05). Functional status did not change and tramadol intake did not differ between patient groups. In controls, no changes occurred in both groups.

**Conclusions:**

Etanercept monotherapy ameliorates disease activity in GC naïve patients with PMR. However, the effect is modest, indicating a minor role of TNF-α in PMR.

**Trial registration:**

ClinicalTrials.gov (NCT00524381).

## Introduction

Polymyalgia rheumatica (PMR) is the most common chronic inflammatory disease in the elderly [1]. Clinically, it is characterized by pain in the neck and lower back as well as proximal extremity complaints, including tender, aching, and stiff muscles. Patients feel fatigued and their level of physical activity is reduced [1]. These symptoms are accompanied by elevated erythrocyte sedimentation rate (ESR) and increased blood levels of C-reactive protein (CRP) [1].

The knowledge of the etiology and pathophysiology of PMR is modest. The prevailing view is that PMR reflects inflammatory processes in synovial membranes in joints, bursae, and tendons [1-4]. Several studies have found elevated blood levels of various proinflammatory cytokines in PMR [5]. Recently, we showed that levels of proinflammatory cytokines, including IL-6 and TNF-α, which both potently induce the expression of acute phase reactants [6] and promote nociception [7,8], are increased in the interstitium of affected muscles [9].

The only effective treatment is medium-dose glucocorticoids (GC), which abolish symptoms within a few days [10]. However, because long-term treatment is necessary, serious adverse effects, including type 2 diabetes, hypertension, and osteoporosis, are frequent [11].

In patients with rheumatoid arthritis (RA), another chronic inflammatory disease and an important differential diagnosis in PMR [10,12,13], administration of TNF-α inhibitors has been a therapeutic success [14]. In PMR, however, no effect of the TNF-α antagonist infliximab on relapse frequency and use of prednisone was found in a recent randomized controlled trial (RCT) of newly diagnosed patients [15]. Still, it should be noted that seven clinics participated in that study [15]. It can be expected that a high number of clinics and doctors involved increases the variation associated with clinical evaluation and decisions concerning patients and, accordingly, decreases the ability to detect differences between treatment with TNF-α blockade and placebo. Furthermore, in the mentioned RCT, patients had successfully been treated with prednisone for some weeks before the start of infliximab therapy, which was applied in parallel with a fixed tapering of prednisone treatment [15]. If at all stages the scheduled prednisone dose *per se *would be sufficient to control the disease, this may have hampered the ability of the study to detect any potential beneficial effect of the added TNF-α blockade.

Finally, even if infliximab has no effect in patients with PMR, the TNF-α antagonist etanercept might still be effective, because the two TNF-α inhibitors act by different mechanisms, being an anti-TNF-α monoclonal antibody and a soluble recombinant Fc-coupled TNF-α receptor fusion protein, respectively. Correspondingly, infliximab and etanercept have different therapeutic potentials in other diseases, for example, only infliximab is effective in granulomatosis disorders such as Crohn's disease and Wegener's granulomatosis [16]. Also, small uncontrolled studies have pointed to a beneficial effect of etanercept in patients with PMR [17,18]. Moreover, in a RCT of patients with giant cell arteritis (GCA), which is intimately related to PMR, etanercept was shown to be an effective therapy [19].

As there is a need for effective drugs other than GCs for PMR, and because existing evidence does not exclude a role of etanercept, in the present study we performed the first RCT of etanercept in patients with PMR. The study was a parallel group in a placebo-controlled, double-blinded, RCT with etanercept in a group of newly diagnosed, GC naïve PMR patients and non-PMR control subjects. The trial was carried out at a single center, and all patients were diagnosed and evaluated by the same chief rheumatologist. Furthermore, etanercept was the only anti-inflammatory drug administered. So, the study allows evaluation of the pure effect of etanercept treatment in patients with PMR without any possible blurring interference due to, for example GC treatment. The duration of the trial was 14 days, because for etanercept to be an attractive alternative to GCs in the treatment of patients with PMR, beneficial effects must occur rapidly. Rapid action of an effective anti-TNF-α therapy can also be expected, if TNF-α does in fact play a key role in the pathophysiology of PMR. In agreement with this view, in RA a clear effect of TNF-α antagonism can be expected within a week [20].

## Materials and methods

### Participants

Patients with suspected PMR were recruited by referral from general practitioners in the Copenhagen municipal area from July 2007 to May 2009. Non-PMR control subjects were recruited by newspaper advertising during the same period. Patients were eligible if they had been diagnosed with PMR according to the Chuang criteria [21], and if they did not fulfill the exclusion criteria, which were: prior or current use of GCs or other immunosuppressive drugs; signs of GCA including cranial symptoms of vasculitis (headache, visual disturbances, jaw claudication, abnormal pulsation or wall of temporal artery, scalp tenderness); infections with systemic impact; hepatitis B or C infection; positive tuberculosis screening tests (thorax x-ray imaging, Mantoux skin test and Quantiferon tuberculosis blood test); positive blood or urine culture; uncontrolled diabetes mellitus; uncontrolled hypertension; severe heart failure (New York Heart Association class 3 and 4); other inflammatory diseases than PMR; cancer in the past five years; neuromuscular disease; thyroid disease; disturbance of calcium homeostatis. Control subjects fulfilled the same exclusion criteria as patients and were matched according to sex, age, and body mass index (BMI). Withdrawal criteria were non-compliance, serious exacerbation of the disease, development of serious infections, development of heart failure, and other suspected unexpected serious adverse reactions.

Concurrent use of nonsteroidal anti-inflammatory drugs and GCs was not allowed in both patients and control subjects. One hundred 50 mg tablets of the centrally active opioid-like analgesic tramadol (Mandolgin, Sandoz A/S, Odense, Denmark) were given to the patients, who were instructed to administer them in order to adequately control pain symptoms during the trial (no more than eight tablets per day); patients registered their use of tramadol using a standardized form during the entire trial.

### Trial design

The study was conducted at the Department of Rheumatology at Bispebjerg Hospital, Copenhagen, Denmark as a 14-day single-center, double-blinded, prospective RCT, comparing the effect of 14 days of etanercept treatment (n = 10) against 14 days of placebo treatment (n = 10) in a group of 20 patients with PMR and in an equal sized group of matched control subjects.

The trial was approved by the Danish Medicines Agency (approval number 2612-3497), the Ethical Committee (Institutional Review Board, approval number H-D-2007-0040), and by the Danish Data Protection Agency. Furthermore, the trial was entered in the European Eudract database (Eudract number 2007-003009-28), and it was registered in the public database clinicaltrials.gov (trial identifier NCT00524381). The study was conducted according to the International Conference on Harmonisation of Good Clinical Practices and was monitored by a good clinical practice monitoring unit before, during, and after the trial period. At the time of screening for trial inclusion, potential participants received thorough written and oral information of the purpose and duration of the trial as well as of predictable adverse events. Before inclusion in the study, all participants signed a written informed consent.

### Randomization and blinding

Following enrollment by a rheumatology specialist (the senior author), participants were randomly assigned to treatment with etanercept or placebo in a 1:1 ratio. A five-block randomization scheme was generated using the web site Randomization.com[22] by two trial-associated senior nurses, who were also responsible for drug preparation and who had no contact with the participants. When included in the trial, subjects were consecutively assigned an identification number according to the randomization scheme by the same two nurses. Subjects who withdrew from the trial were consecutively replaced by new subjects and allocated to the same treatment. Physicians and technicians in direct contact with participants or those responsible for data and plasma analysis including staff that administered the medication were blinded to group assignment. The blinding code was not broken until all trial outcomes had been collected.

### Examinations

One to three days after inclusion in the study, subjects were examined the first of two times, 14 days apart. They were brought by taxi to the laboratory after an overnight fast including abstinence from alcohol and tobacco. Subjects were allowed to take their usual medication, if any, in the morning before the examination but abstained from analgesics. A cannula was inserted into a forearm vein, and subjects rested in a chair 15 minutes before blood samples were drawn. A clinical examination focusing on joint mobility and muscle tenderness was carried out, and a health assessment questionnaire (HAQ; evaluated for use in RA [23]) and some supplementary questions about morning stiffness and daily physical activity were answered. At the end of the first examination, subjects had their first of four injections with trial medication. The last injection was given three days before the final examination (end of study).

### Interventions

Etanercept (Enbrel, Wyeth Pharmaceuticals New Lane, Hampshire, UK) was injected subcutaneously biweekly in the thigh at the standard dose of 25 mg (1 ml). Placebo was 1 ml isotonic saline. Individual injections were given at least 3 cm apart. To ensure proper blinding, etanercept and placebo, which were both colorless solutions, were prepared in indistinguishable syringes by nurses, who had no interaction with the subjects.

### Study outcomes

The primary outcome was the change in PMR activity score (PMR-AS [24-26]), from baseline to end of study (day 15) in patients and control subjects treated with etanercept or placebo. PMR-AS was calculated from measurements of plasma CRP levels (mg/dl), the duration of morning stiffness (MST, minutes), the ability to raise the arms (E, 3 to 0: 3 = no elevation possible; 2 = elevation possible below shoulder height; 1 = elevation possible above shoulder height; 0 = full elevation possible), physician's global assessment (physician's visual analog scale (VAS_ph_); 0 to 10 cm), and the patients' assessment of pain (patient's visual analog scale (VAS_p_); 0 to 10 cm), as [24]:

$$\begin{array}{l} \text{PMR}-\text{AS}=\text{CRP}\left(\text{mg}/\text{dl}\right)+\text{E}\left(\text{3}-0\right)+\left(\text{MST}\left(\text{min}\right)\times 0.\text{1}\right)+{\text{VAS}}_{ph}\left(0\text{ to 1}0\text{ cm}\right)+{\text{VAS}}_{p}\\ \left(0\text{ to 1}0\text{ cm}\right). \end{array}$$

Secondary outcomes were: changes in ESR, cumulative intake of tramadol during the study, and plasma TNF-α and IL-6 concentrations in all groups at baseline and at the end of the study (day 15). After treatment in patients receiving etanercept, TNF-α concentrations were also measured in immunoglobulin (Ig) G depleted plasma. In patients, functional status was assessed before and after etanercept treatment using the HAQ [23].

To ensure high accuracy of outcome assessments and measurements, all participating staff carefully reviewed the study protocol prior to the start of the trial. Moreover, standard operation protocols were used during all trial-related activities.

### Safety and adverse events

Assessment of the subjects' safety included registration of all adverse events as well as careful examination and questioning of subjects before each injection and/or examination.

### Plasma samples

Blood samples were drawn in stock Vacutainers (Becton Dickinson, Brpndby, Denmark) with EDTA anticoagulant and the proteolysis inhibitor Trasylol (Bayer AG, Leverkusen, Germany). Plasma was harvested by centrifugation at 1,200 rpm at 4°C for 15 minutes and immediately frozen at -80°C until analysis.

### Analytical methods

ESR and blood CRP were measured at an ISO-certified (ISO 15189:2008) clinical laboratory using the Westergen method (Becton Dickinson, BD Sedi-15) and colorimetric slide tests (measurement range: 5 to 90 mg/l; intra- and interassay coefficients of variation (CV): 2.2% and 10.0%, respectively), respectively. Plasma TNF-α and IL-6 were analyzed using the Luminex 100 platform (Ramcon, Birkeroed, Denmark); specific kits used were Milliplex (Millipore, ElectraBox Aps, Roedovre, Denmark). Detection levels and intra- and interassay CVs were: TNF-α 0.05 pg/ml, 3.5% and 3.8%, respectively; IL-6 0.79 pg/ml, 13.6% and 13.3%, respectively. Analyses were carried out on unprocessed plasma. Furthermore, TNF-α was also determined in plasma processed by protein G-based spin columns (Albumin and IgG Depletion Spin Trap, GE Healthcare, Uppsala, Sweden), which remove IgGs including free etanercept and etanercept-TNF-α complexes. To compensate for loss in the columns of free TNF-α and of proteins influencing standard curves, standards dissolved in plasma analogue (matrix included in the MilliPlex kit) were also passed through the spin columns.

### Statistics

We estimated the sample size based on an effect size on PMR-AS of ρ = |0.75|. Thus, at an α-level of 0.05 and at a statistical power of 80% (in two-tailed testing), the minimum sample size should be nine subjects in each treatment arm.

Statistical analysis was performed using SPSS 17.0.1 for Mac (SPSS Inc., Chicago, IL, USA). Unless otherwise stated, data are mean ± standard error of the mean. A two-way analysis of variance was used to determine if data differed between patients and control subjects, if changes occurred with treatment, and if there was any interaction between group and treatment. *P*-values below 0.05 in two-tailed testing were considered significant.

## Results

**Figure 1:**
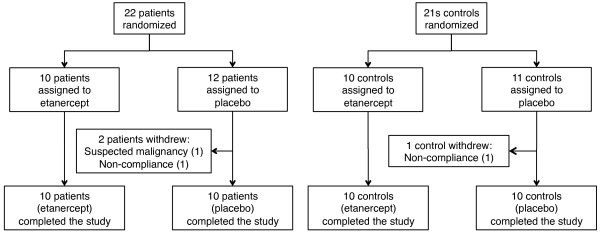
**Flow of patients and controls through the trial**. This includes randomization, withdrawal numbers and reasons, and number of participants, who completed the study.

**Figure 2:**
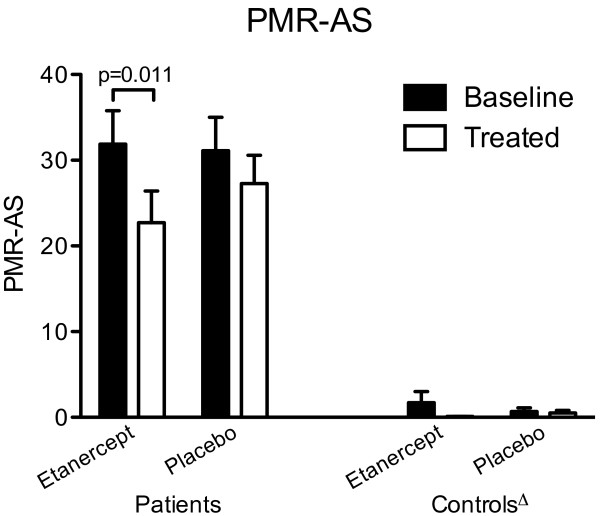
**Primary outcome**. Polymyalgia rheumatica activity score (PMR-AS) in 20 patients with PMR and 20 non-PMR control subjects at baseline (black bars) and after (white bars) 14 days of treatment with etanercept or placebo. PMR-AS is calculated from the blood level of C-reactive protein, duration of morning stiffness, subject's assessment of pain (visual analog score (VAS)), physician's global assessment (VAS), and the subject's ability to elevate the arms (see text). Δ all values in control subjects significantly different (*P *< 0.0001 to 0.01) from values in patients.

**Figure 3:**
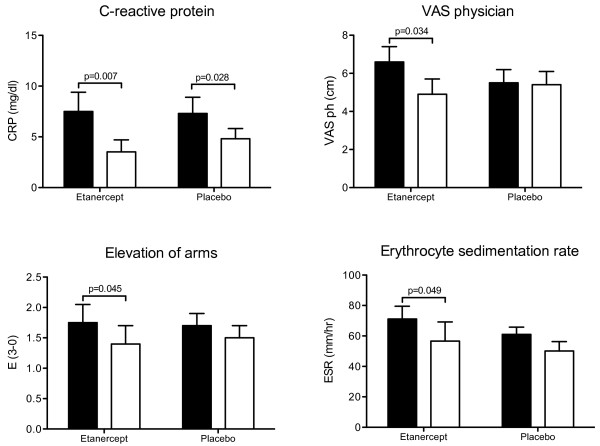
**Clinical and paraclinical measurements in patients with polymyalgia rheumatica at baseline (black bars) and after (white bars) 14 days of treatment with etanercept or placebo**. C-reactive protein (CRP), patients' ability to elevate the arms (E, 3 to 0: 3 = no elevation possible; 2 = elevation possible below shoulder height; 1 = elevation possible above shoulder height; 0 = full elevation possible), the physician's global assessment (visual analog score, VAS), and erythrocyte sedimentation rate (ESR).

Twenty-two patients with PMR were included in the trial; of these, two patients, both receiving placebo, withdrew from the study (Figure 1). Twenty-one control subjects without PMR were included; one control subject receiving etanercept withdrew (Figure 1). Baseline anthropometric and clinical findings did not differ (*P *> 0.05) between patients who received etanercept and patients who received placebo (Figures 2 and 3, and Table 1). Nor did these variables differ between control subjects receiving etanercept and placebo, respectively (Table 2, *P *> 0.05). Age, BMI, and blood pressure did not differ (*P *> 0.05) between patients and control subjects; before treatment all other measurements differed significantly (*P *< 0.001 to 0.01) between these groups (Tables 1 and 2, and Figure 3).

**Table 1:** Characteristics of patients at baseline and after etanercept/placebo treatment

	Before etanercept (n = 10)	Before placebo (n = 10)	After etanercept (n = 10)	After placebo (n = 10)
**Sex (female/male)**	6/4	7/3	-	-
**Age (years)**	72.6 ± 2.6	71.4 ± 3.6	-	-
**BMI (kg/m^2^)**	23.6 ± 3.4	24.9 ± 4.1	-	-
**Blood pressure** **(systolic/diastolic, mmHg)**	160 ± 5/87 ± 4	159 ± 4/87 ± 5	-	-
**Smokers (n)**	2	3	-	-
**Hypertension (n)**	6	3	-	-
**Hypercholesterolemia (n)**	3	2	-	-
**Physical activity level** **before PMR onset** **(1, high; 2, medium; 3, low)**	1.4 ± 0.1	1.9 ± 0.2	-	-
**Physical activity level** **(1, high; 2, medium; 3, low)**	2.9 ± 0.1	2.9 ± 0.1	2.7 ± 0.2	3.0 ± 0
**Morning stiffness (min)**	98 ± 16	117 ± 19	70 ± 17	103 ± 21
**Patients' assessment of pain, VAS (0-10 cm)**	6.2 ± 0.7	5.0 ± 0.8	5.9 ± 0.6	5.4 ± 0.5
**Cumulative intake of tramadol (tablets/14 days)**	-	-	47 ± 8	55 ± 12

Data are mean ± standard error of the mean. PMR, polymyalgia rheumatica; VAS, visual analog scale.

**Table 2:** Characteristics of control subjects at baseline and after etanercept/placebo treatment

	Before etanercept (n = 10)	Before placebo (n = 10)	After etanercept (n = 10)	After placebo (n = 10)
**Sex (female/male)**	9/1	6/4	-	-
**Age (years)**	69.9 ± 1.2	77.0 ± 5.7	-	-
**BMI (kg/m^2^)**	25.5 ± 0.9	26.4 ± 1.4	-	-
**Blood pressure** **(systolic/diastolic, mmHg)**	148 ± 5/85 ± 3	150 ± 4/85 ± 2	-	-
**Smokers (n)**	2	3	-	-
**Hypertension (n)**	5	4	-	-
**Hypercholesterolemia (n)**	2	1	-	-
**Physical activity level** **(1, high; 2, medium; 3, low)**	*1.5 ± 0.2	*1.9 ± 0.1	*1.6 ± 0.2	*1.8 ± 0.2
**ESR (mm/hr)**	*11.0 ± 3.1	*9.1 ± 1.3	*9.4 ± 2.8	*13.5 ± 1.2
**CRP (mg/dl)**	*1.1 ± 0.1	* < 1.0	* < 1.0	* < 1.0
**Elevation of arms (3-0)**	*0	*0	*0	*0
**Morning stiffness (min)**	*6.0 ± 2.0	*0	*0	*0
**Physician's global assessment, VAS (0-10 cm)**	*0.2 ± 0.2	*0	*0	*0
**Subjects' assessment of pain, VAS (0-10 cm)**	*0.75 ± 0.5	*1.0 ± 0.4	*0.06 ± 0.1	*0

Data are mean ± standard error of the mean.

CRP, C-reactive protein; ESR, erythrocyte sedimendation rate; VAS, visual analog scale.

*Values significantly different from values in patients with polymyalgia rheumatica (*P *< 0.001 to 0.01).

After the 14 days of trial participation, all patients were treated with 20 mg/day prednisolone. Within a week, prednisolone abolished any remaining PMR symptoms and normalized CRP and ESR in all patients, supporting the PMR diagnosis.

### Primary outcome: PMR-AS

At baseline, PMR-AS did not differ between patients treated with etanercept and patients treated with placebo (Figure 2, *P *> 0.05); it was, however, significantly higher in patients than in control subjects (Figure 2, *P *< 0.0001 to 0.01). In all control subjects, PMR-AS did not differ significantly from zero (Figure 2, *P *> 0.05).

During the 14 days of etanercept treatment, PMR-AS significantly decreased by 24% (95% confidence interval: 12 to 33%) in patients (Figure 2), but remained significantly higher in patients compared with control subjects (Figure 2, *P *< 0.0001 to 0.01). In patients receiving placebo, PMR-AS did not change (*P *> 0.05).

### Secondary outcomes

**Figure 4:**
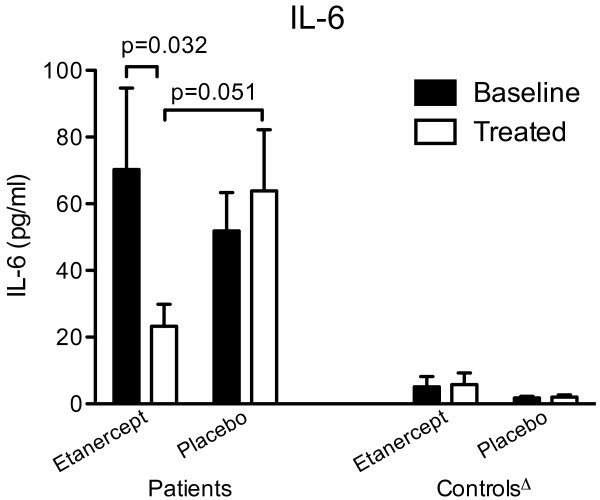
**Plasma IL-6 concentrations in patients with polymyalgia rheumatica (PMR) and non-PMR control subjects at baseline (black bars) and after (white bars) 14 days of treatment with etanercept or placebo**. Δ all values in control subjects significantly different (*P *< 0.01 to 0.001) from values in patients.

In patients with PMR, CRP, E and VAS_ph _decreased significantly during etanercept treatment (Figure 3), whereas during placebo treatment only CRP decreased and to a lesser extent (*P *< 0.05; Figure 3). Also the other components of the PMR-AS (MST and VAS_p_) tended to decrease during etanercept treatment; however, the changes were not statistically significant (Table 1, *P *> 0.05). ESR (Figure 3) as well as IL-6 (Figure 4), which at baseline were markedly higher in patients than in control subjects, decreased in PMR patients receiving etanercept, but not (*P *> 0.05) in patients receiving placebo. The cumulative intake of tramadol (Table 1) was 17% lower in patients treated with etanercept compared with placebo, but the difference was not significant (*P *> 0.05). HAQ variables did not change in patients receiving etanercept or placebo (data not shown). By the end of the trial, the various variables still did not differ significantly between patients receiving etanercept and placebo (*P *> 0.05). However, the mean IL-6 concentration was markedly higher in patients receiving placebo, and this difference was of borderline significance (Figure 4). Furthermore, all disease parameters were still significantly higher compared with values in control subjects (*P *< 0.05). In control subjects, no changes were seen after either etanercept or placebo treatment.

**Figure 5:**
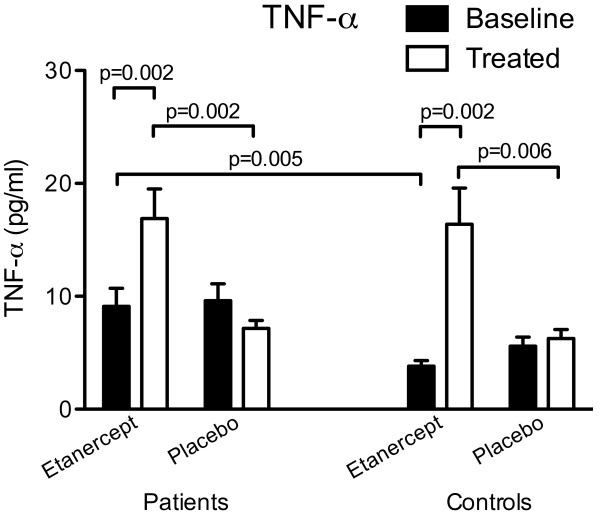
**Plasma TNF-α concentrations in patients with polymyalgia rheumatica (PMR) and non-PMR control subjects at baseline (black bars) and after (white bars) 14 days of treatment with etanercept or placebo**.

At baseline, plasma TNF-α concentrations were significantly higher in patients than in control subjects (Figure 5). During etanercept treatment TNF-α concentrations markedly increased in both groups (Figure 5). During placebo treatment, TNF-α concentrations in plasma decreased in patients but stayed constant in control subjects (Figure 5). The high concentrations seen in patients treated with etanercept were reduced by 61% (*P *= 0.02) by IgG depletion of plasma, and became similar to concentrations in placebo-treated patients (values before vs after IgG depletion in etanercept (n = 10) and placebo (n = 10) treated patients, respectively: 16.9 ± 2.6 pg/ml vs 10.4 ± 1.3 pg/ml; 7.1 ± 0.7 pg/ml vs 11.5 ± 1.3 pg/ml).

### Safety and adverse events

Generally, etanercept treatment was well tolerated in all subjects. Two patients and one control subject, all treated with etanercept, had minor local injection-site reactions (rashes). One control subject treated with placebo reported an unsuspected feeling of fatigue. No suspected unexpected serious adverse reactions were observed in any of the subjects.

## Discussion

The present study is the first RCT of the effect of etanercept in patients with PMR. The major finding is that 14 days of etanercept monotherapy ameliorates disease activity in newly diagnosed, GC-naïve patients with PMR. However, the effect is modest indicating that TNF-α does not have a predominant role in the pathophysiology of PMR.

The positive effect of etanercept is evident from the 24% reduction of PMR-AS in patients treated with the drug, as well as the insignificant changes in PMR-AS observed in placebo-treated patients (Figure 2). The reduction of PMR-AS in patients treated with etanercept reflected decreases in all paraclinical as well as objective and subjective clinical components of PMR-AS. Among these, statistically significant improvements in shoulder mobility (E), physician's global assessment (VAS_ph_) and CRP were seen, and these changes were paralleled by a significant decrease in ESR and plasma IL-6 concentration (Figures 3 and 4). The fact that both etanercept and placebo had no effect in non-PMR control subjects (Figures 2 to 4, and Table 2) shows that the significant effects of etanercept seen in patients with PMR were indeed disease specific.

However, the view that the effect of etanercept is modest in patients with PMR is suggested by the fact that the reductions in both MST and VAS_p _were not statistically significant (Table 1). Furthermore, at the end of the 14 days of etanercept treatment, PMR-AS was still not only higher than in control subjects but also not significantly different from values in placebo-treated patients (Figure 2). Correspondingly, although the tramadol intake was lower in etanercept compared with placebo-treated patients, the difference was not statistically significant, and functional status as evaluated by HAQ did not improve in any of the groups.

In order to verify that the anti-TNF-α treatment was still effective at the final evaluation by day 15, that is, three days after the last etanercept injection, and that potential effects of the blockade accordingly were not underestimated, plasma TNF-α concentrations were determined. However, instead of being reduced, in etanercept-treated patients the TNF-α concentrations were higher than before treatment and also higher than in placebo-treated patients (Figure 5). The increased TNF-α values observed after etanercept treatment were probably due to the simultaneous detection of both free and accumulated etanercept-bound TNF-α [27]. In agreement with this view, during etanercept treatment of control subjects TNF-α concentrations were also markedly increased (Figure 5).

Furthermore, in patients treated with etanercept, TNF-α concentrations were reduced by 61% after IgG depletion of plasma. This process also removes etanercept (free etanercept as well as etanercept-TNF-α complexes), which contains the Fc fragment of IgG1. TNF-α concentrations were similar in IgG-depleted plasma from etanercept-treated compared with placebo-treated patients. As some etanercept-bound TNF-α will remain in IgG-depleted plasma from etanercept-treated patients, while only free TNF-α is present in IgG-depleted plasma from placebo-treated patients, the similar overall TNF-α concentrations in IgG-depleted plasma most likely reflect that free TNF-α concentrations were lower in plasma from etanercept-treated patients than in plasma from placebo-treated patients. So, these observations indicate that by the end of the trial, etanercept was still present in plasma and able to lower free TNF-α concentrations compared with values in placebo-treated patients [27].

In the present study, TNF-α concentrations in plasma were higher in patients with PMR than in non-PMR controls subjects (Figure 2) before treatment in both treatment groups. This is in accordance with findings in a recent study, in which we showed that interstitial TNF-α concentrations in affected muscle are increased in untreated PMR and normalized in parallel with complete clinical remission during 14 days of prednisolone therapy [9]. Although these findings are suggestive, the fact that in the present study TNF-α blockade had only a modest limiting effect on disease activity indicates that TNF-α is not a major determinant of the pathophysiology in PMR. Interestingly, in a study of two patients with previously untreated GCA, a condition generally considered to be closely related to PMR, a dramatic improvement was observed within 14 days of monotherapy with the anti-TNF-α monoclonal antibody infliximab, and it was concluded that in this disease TNF-α plays a major role in mediating inflammation [28].

In the present study, the same experienced rheumatology specialist evaluated all patients. It is interesting to note that although being blinded to treatment allocation, the specialist's evaluation of disease activity as reflected by shoulder mobility (E) and VAS_ph_, was in better agreement with paraclinical measures (ESR, CRP, and IL-6) than the patients' own evaluations of disease activity as apparent from MST, VAS_p_, and HAQ. Due to the monotherapeutic design of the present RCT, potential effects of etanercept were not obscured by simultaneous administration of other anti-inflammatory drugs such as GCs. As a consequence, however, the trial had to be brief. Thus, it is possible that a larger effect of etanercept would have been demonstrated had it been possible to extend the duration of the trial. However, in patients with RA the effect of etanercept occurs rapidly [20]. Furthermore, in patients with GCA a small uncontrolled study showed a dramatic effect of infliximab monotherapy treatment in the early phase [28]. Conversely, a RCT has shown that extension of infliximab therapy to 22 weeks does not diminish the need for simultaneous GC therapy [29]. Still, in some contrast to the latter study, a GC-sparing effect of etanercept has been demonstrated in a recent RCT when administered late in the course of GCA in patients with side effects from GC [19]. However, such patients may have a particularly strong inflammatory reaction making them relatively GC-resistant [6,30] and sensitive to TNF-α blockade [31].

The disease ameliorating effect of etanercept seen in the present study contrasts with the lack of benefit of infliximab found in a RCT with GC-treated, newly diagnosed patients with PMR [15]. This discrepancy may reflect differences in study design or differences in mechanism of action of the two TNF-α antagonists. Arguing in favor of the latter possibility, in a small, uncontrolled study, it was demonstrated that etanercept was beneficial in the treatment of GC-treated patients with newly diagnosed PMR and decompensated diabetes [18]. However, arguing against the latter possibility, in line with our findings, infliximab monotherapy induced remission in a small, uncontrolled study with newly diagnosed patients with PMR [17]. Moreover, in another uncontrolled study of a small number of patients with longstanding, GC-resistant PMR, administration of infliximab was accompanied by diminished disease activity [32].

Overall, current evidence indicates that the effect of TNF-α blockade in PMR and GCA is moderate. For that reason and considering the high cost, TNF-α therapy in PMR and GCA seems justified solely when these diseases can only be controlled by GC doses that are untenable due to serious adverse effects and comorbidities induced or worsened by steroids.

## Conclusions

In conclusion, the present RCT has demonstrated that etanercept monotherapy ameliorates disease activity in newly diagnosed, GC-naïve patients with PMR. However, the effect is modest, indicating that TNF-α does not play a central role in the pathophysiology of PMR.

## Abbreviations

BMI: body mass index; CRP: C-reactive protein; CV: coefficient of variation; ESR: erythrocyte sedimentation rate; GC: glucocorticoid; GCA: giant cell arteritis; HAQ: health assessment questionaire; Ig: immunoglobulin; IL: interleukin; MST: morning stiffness; PMR: polymyalgia rheumatica; PMR-AS: PMR activity score; RA: rheumatoid arthritis; RCT: randomized controlled trial; TNF: tumor necrosis factor; VAS: visual analog score; VAS_p_: patient's visual analog score; VAS_ph_: physician's visual analog score.

## Competing interests

The authors declare that they have no competing interests.

## Authors' contributions

FK and HG contributed equally to the planning and conduct of the trial as well as to the analysis and interpretation of study outcomes. Both authors drafted and approved the manuscript.
